# Supplementary material for: Splice-Junction-Based Mapping of Alternative Isoforms in the Human Proteome
Source: Cell Rep. Author manuscript; Available in PMC 2020 Jan 15. (PMC6961840; doi:10.1016/j.celrep.2019.11.026)
Supplement: 2 [file NIHMS1546469-supplement-2.zip › DF1/PXD010154/Testis/Testis_31_EPB41L3_QEDAPMIEPLVPEEVSSQK.pdf]

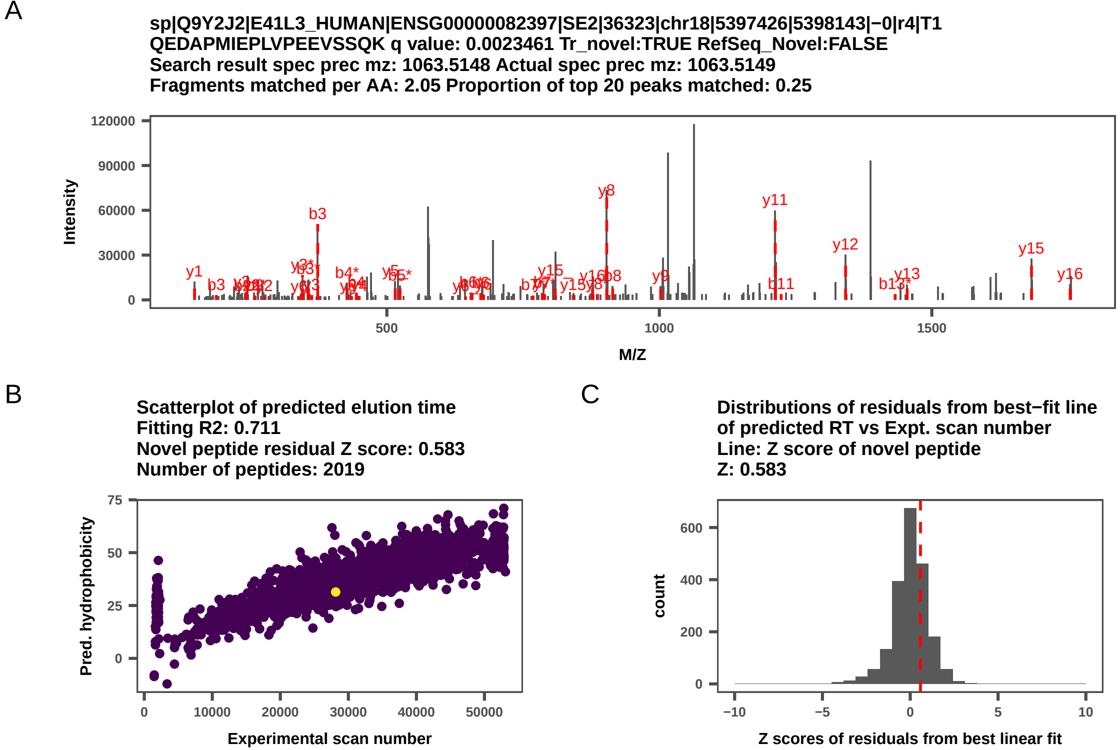
A
sp|Q9Y2J2|E41L3_HUMAN|ENSG00000082397|SE2|36323|chr18|5397426|5398143|−0|r4|T1
QEDAPMIEPLVPEEVSSQK q value: 0.0023461 Tr_novel:TRUE RefSeq_Novel:FALSE
Search result spec prec mz: 1063.5148 Actual spec prec mz: 1063.5149
Fragments matched per AA: 2.05 Proportion of top 20 peaks matched: 0.25
Intensity
120000
90000
60000
30000
0
500
1000
1500
M/Z
y1
b3
y8
y11
y12
y15
y16
y9
b11
b4*
y13
b13*
B
Scatterplot of predicted elution time
Fitting R2: 0.711
Novel peptide residual Z score: 0.583
Number of peptides: 2019
Pred. hydrophobicity
75
50
25
0
0
10000
20000
30000
40000
50000
Experimental scan number
C
Distributions of residuals from best−fit line
of predicted RT vs Expt. scan number
Line: Z score of novel peptide
Z: 0.583
count
600
400
200
0
−10
−5
0
5
10
Z scores of residuals from best linear fit
